# Biochemical, Sensory, and Molecular Evaluation of Flavour and Consumer Acceptability in Australian Papaya (*Carica papaya* L.) Varieties

**DOI:** 10.3390/ijms23116313

**Published:** 2022-06-05

**Authors:** Ziwei Zhou, Ido Bar, Rebecca Ford, Heather Smyth, Chutchamas Kanchana-udomkan

**Affiliations:** 1 Centre for Planetary Health and Food Security, School of Environment and Science, Griffith University, Nathan, QLD 4111, Australia; ziwei.zhou2@griffithuni.edu.au (Z.Z.); i.bar@griffith.edu.au (I.B.); c.kanchana-udomkan@griffith.edu.au (C.K.-u.); 2 Centre for Nutrition and Food Sciences, Queensland Alliance for Agriculture and Food Innovation, The University of Queensland, St. Lucia, Brisbane, QLD 4072, Australia; h.smyth@uq.edu.au

**Keywords:** papaya flavour, volatiles, sweetness, sensory descriptive analysis, consumer acceptability, gene expression

## Abstract

Inconsistency in flavour is one of the major challenges to the Australian papaya industry. However, objectively measurable standards of the compound profiles that provide preferable taste and aroma, together with consumer acceptability, have not been set. In this study, three red-flesh papayas (i.e., ‘RB1’, ‘RB4’, and ‘Skybury’) and two yellow-flesh papayas (i.e., ‘1B’ and ‘H13’) were presented to a trained sensory panel and a consumer panel to assess sensory profiles and liking. The papaya samples were also examined for sugar components, total soluble solids, and 14 selected volatile compounds. Additionally, the expression patterns of 10 genes related to sweetness and volatile metabolism were assessed. In general, red papaya varieties had higher sugar content and tasted sweeter than yellow varieties, while yellow varieties had higher concentrations of citrus floral aroma volatiles and higher aroma intensity. Higher concentrations of glucose, linalool oxide, and terpinolene were significantly associated with decreased consumer liking. Significant differences were observed in the expression profiles of all the genes assessed among the selected papaya varieties. Of these, *cpGPT2* and *cpBGLU31* were positively correlated to glucose production and were expressed significantly higher in ‘1B’ than in ‘RB1’ or ‘Skybury’. These findings will assist in the strategic selective breeding for papaya to better match consumer and, hence, market demand.

## 1. Introduction

Papaya (*Carica papaya* L.) is a tropical fruit crop cultivated worldwide. In Australia, papaya was first introduced as a commercial crop in the 1900s, with currently >90% of production in the tropical regions of North Queensland [1]. Since its introduction, Australian papaya production has steadily increased, with 6.3 thousand tonnes produced in 2020 [2]. Initial breeding efforts for Australian papaya have focused on traits such as resistance to ringspot virus and increased yield rather than flavour or other consumer-preferred characteristics [3]. However, the commercial Australian papaya varieties vary greatly in their flavour profiles, likely impacting consumer demand and, hence, sales. These range from sweet and fruity to others with bitter aftertastes and unpleasant aromas [1]. Therefore, a major objective of the national papaya breeding program (PP18000, funded by Hort Innovation) is to develop varieties with improved flavours.

Flavour is a multifactorial trait that includes both mouth perception and aroma [4], contributed by the interactions between sugar, organic acids, and volatile organic compounds (VOCs) [3,5]. Several studies have illustrated that combinations of different amounts and ratios of sugar types and VOCs directly impact papaya consumer liking [3,6,7,8]. Therefore, the underpinning biochemical compounds and their expression levels are key selection targets for improvement towards preferred flavours. Accordingly, attempts have been undertaken over the past 50 years to uncover the key discriminant compounds that produce the unique aromas and flavours of papaya. Methods have included headspace, odour olfactometry, and gas chromatography mass spectrometry (GCMS) technology, resulting in the discovery of more than 400 VOCs that are proposed to be related to papaya aroma and flavour. The accurate identification and quantification of the VOCs was reliant on reference standards, of which a limited and varying number were used [4,6,8,9,10,11]. In previous studies, papaya aroma and flavour compounds have generally fallen into ester- or terpene-rich chemotypes, with high concentrations of linalool and benzyl isothiocyanate commonly detected [6,8,9,12,13]. Meanwhile, papaya sweetness is largely derived from three soluble sugars: glucose, fructose, and sucrose [4]. During fruit ripening, glucose and fructose accumulate and are converted to sucrose under multiple enzyme metabolisms, and sucrose is the predominant sugar that contributes to flesh sweetening [14]. To evaluate papaya sweetness, it is important to assess the total sugar content and the percentage of each type of soluble sugar present [15]. In addition to the precise evaluation of biochemical metabolites involved in aroma and flavour production, sensory panel testing and consumer acceptance surveying are commonly used to determine flavour attributes preferred by consumers [8,16,17]. Blind preference surveying may reveal the range of consumer liking levels among diverse papaya samples. The outputs may then be used to assess for correlation with biochemical profiles to identify preferred aromas, flavours and, hence, varieties.

Moreover, genomic-based studies may be undertaken to identify, characterise, and validate sequences and putative alleles related to aroma, flavour, and sweetness. This approach may also aid with understanding the metabolic pathways of the underpinning sugar and VOCs. In a previous study, enzymes involved in the production, translocation, and storage of sugars in papaya fruit included the soluble acid invertase, the insoluble acid invertase, neutral invertase, sucrose phosphate synthase (SPS), and sucrose synthase (SS) [18]. Accordingly, several genes related to these functional enzymes have been identified in previous studies for use in the selection of papaya varieties with higher sugar content [19,20,21]. In addition, the isoprenoid biosynthesis, the shikimic acid, and the acyl lipid catabolism pathways were associated with the production of papaya fruit aroma and flavour VOCs [4]. Further research is required to identify the genes and alleles that encode enzymes and regulatory sequences in these pathways and that govern VOC synthesis.

This study aimed to characterise the flavour profiles of the major commercial papaya varieties in Australia through sensory descriptive profiling, consumer acceptability study, VOC profiling, and sugar component determination. Furthermore, the differential expression of flavour-related gene sequences in papaya was evaluated, and their underpinning metabolic pathways are proposed.

## 2. Results

### 2.1. Sensory Descriptive Profiling

**Figure 1:**
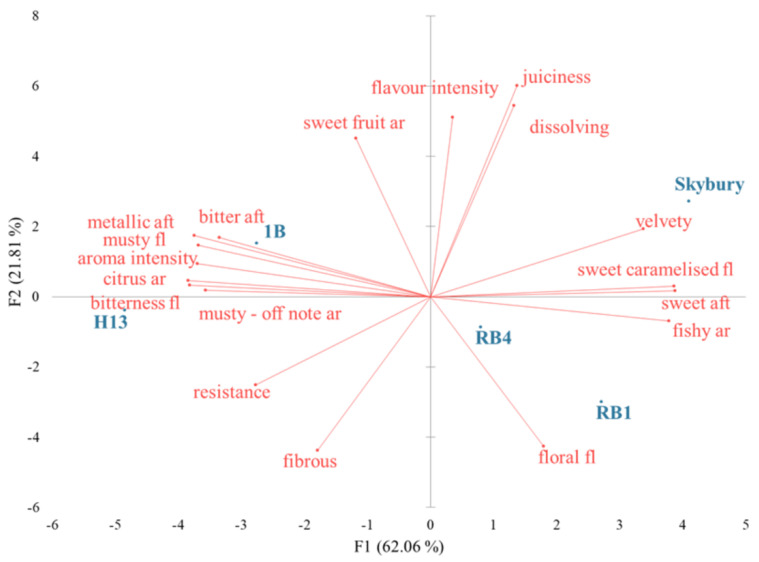
PCA plot of the sensory descriptive data of five papaya varieties (axes F1 and F2: 83.87%). The blue dots represent the five papaya varieties, and the red loading plots show the correlation between sensory descriptors, where ar = aroma, fl = flavour, and aft = aftertaste.

A PCA plot of the papaya sensory descriptive data demonstrated that 83.87% of the variability between sensory attributes and papaya varieties occurred in the first two dimensions (Figure 1). Red papaya varieties ‘RB1’, ‘RB4’, and ‘Skybury’ were clustered together on the right-hand side of the plot and were characterised by high overall *flavour intensity*, a dominating *sweet caramelised* and *floral* flavour as well as a *sweet aftertaste*. The texture was *velvety* with high *dissolving* and *juiciness*. On the contrary, yellow papaya varieties ‘1B’ and ‘H13’, which were clustered together on the left-hand side of the plot, had a strong overall *aroma intensity* with high *citrus* and *sweet fruit* aromas. The texture was *fibrous* and *resistance*. In addition, the yellow varieties had a significant *musty* and *bitter* flavour as well as an intense *bitter aftertaste*.

### 2.2. Sugar Content Determination

The total soluble solids (TSS) values and concentrations of the three main soluble sugars from five papaya varieties (i.e., ‘RB1’, ‘RB4’, ‘Skybury’, ‘1B’, and ‘H13’) are shown in Table 1. Significant differences in TSS levels among the varieties were detected and expressed as ◦Brix. Among them, ‘Skybury’ had the highest Brix (12.8 ± 1.3). The lowest Brix levels were detected in ‘1B’ and ‘H13’, at 9.5 ± 0.7 and 9.2 ± 0.9, respectively. The TSS of ‘RB1’ was lower than ‘Skybury’ but significantly higher than the two yellow papaya varieties. Instead, ‘RB4’, which is also a red papaya variety, was not significantly different from ‘RB1’, ‘1B’, or ‘H13’. The sugar compositions of all samples were not significantly different. However, the percentage of glucose in the total sugar level was statistically different among the five varieties. ‘1B’ (24.1 ± 0.05) and ‘H13’ (24.4 ± 0.03) were significantly higher than ‘Skybury’ (18.4 ± 0.02), illustrating the higher proportion of glucose accumulated in yellow papaya varieties than red.

### 2.3. Volatile Compound Analysis

In total, 14 volatiles were detected from the five papaya varieties assessed (Table 2), comprising 12 monoterpenes and two benzenes. From these, 11 were detected from all five varieties. β-myrcene was detected in all varieties except 1B, and α-pinene and 3-carene were detected in all samples except ‘RB4’ and ‘1B’. The concentrations of linalool oxide, terpinolene, and citral were significantly different between varieties. ‘1B’ had significantly higher concentrations of linalool oxide and terpinolene than the two red papaya varieties ‘RB1’ and ‘Skybury’. The concentrations of citral were significantly different between the two yellow papaya varieties, while ‘1B’ had a 2.5-fold higher concentration of citral than ‘H13’. In addition, higher concentrations of benzenes than monoterpenes were detected in all varieties assessed.

### 2.4. Correlation and Multiple Linear Regression Analyses

The correlations between the consumer-liking scores and sensory descriptors scores are shown in Table 3. In total, 13 sensory attributes were significantly correlated to consumer liking (*p*-value < 0.05). Among these, *floral flavour*, *sweet aftertaste*, *sweet caramelised flavour*, *fishy aroma,* and *velvety texture* were positively correlated, while *aroma intensity*, *musty flavour*, *musty off-note aroma*, *sweet fruit aroma*, *citrus aroma*, *bitter aftertaste*, *bitterness flavour,* and *fibrous texture* were negatively correlated to consumer liking. The flavour-related sensory descriptors (i.e., flavour and aftertaste), which were significantly correlated to consumer-liking scores, were then correlated to fruit sweetness level including the TSS values; concentrations of glucose, fructose, and sucrose; as well as the percentage of glucose and sucrose in the total sugar (Table 4). The aroma-related sensory descriptors with high significance were correlated to the relative concentrations of the volatiles (Table 4). A significant positive correlation (*p*-value < 0.05) was found among the TSS values to the *sweet caramelised flavour* and *sweet aftertaste*, whilst the %Glucose was significantly negatively correlated to these two descriptors. In addition, glucose concentration and the %Glucose were significantly positively correlated to the *bitter aftertaste*, *bitterness flavour*, and *musty flavour*, while the TSS level was negatively correlated to these three attributes. Moreover, %Sucrose was negatively correlated to the *bitterness flavour* and *musty flavour*. The VOCs, linalool, linalool oxide, and terpinolene, were significantly correlated to the selected sensory descriptors. These three volatiles were positively correlated to the *citrus aroma* and *aroma intensity* and negatively correlated to the *fishy aroma*. In addition, linalool oxide and terpinolene were positively correlated to the *musty off-note aroma* (Table 4).

Multiple regression of the significant biochemical variables showed that %Glucose, linalool oxide, and terpinolene, together, were the best predictors of consumer liking. A multiple regression model was generated (*Gl* = %Glucose, *Lo* = Linalool oxide, and *Te* = Terpinolene):

Liking=9.60−15.16Gl+14.64Lo−67.08Te

**Figure 2:**
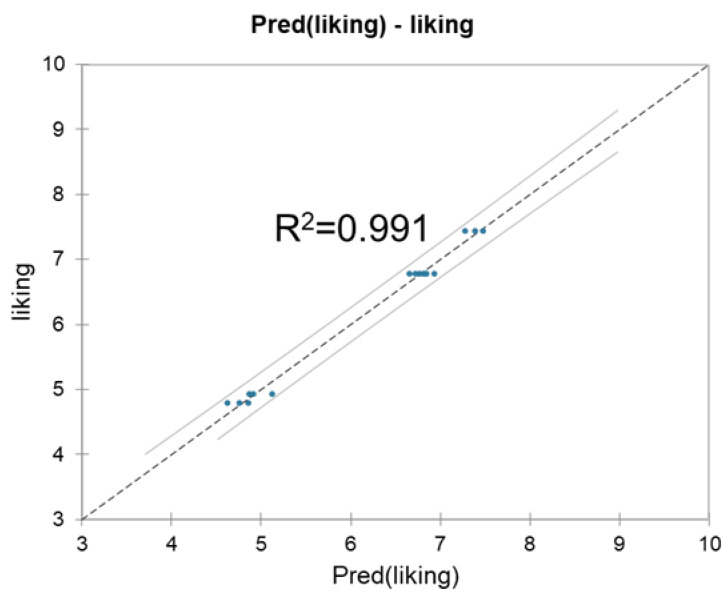
Correlation between actual and predicted mean scores for consumer liking.

The correlation between the observed and predicted liking scores is shown in Figure 2. The coefficient of determination (R^2^) value from the analysis of variance for the regression was 0.991, indicating that over 99% of the variability in consumer liking could be explained by the combination of the percentage of glucose in total sugar together with concentrations of linalool oxide and terpinolene.

### 2.5. Differential Gene Expression Analysis of Flavour-Related Genes

**Figure 3:**
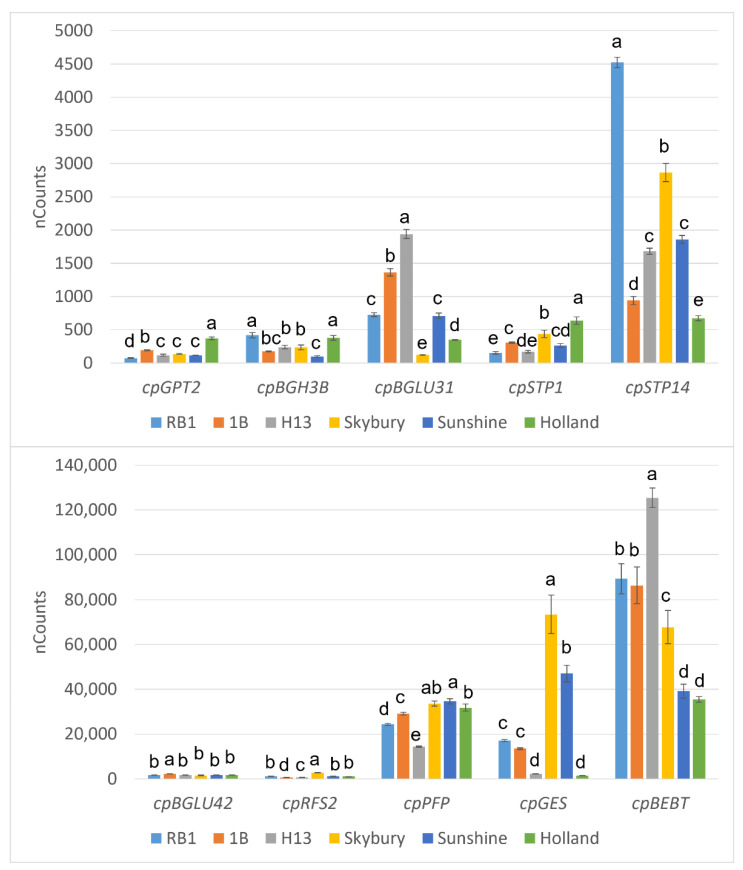
Expression levels of flavour-related genes in papaya varieties based on NanoString analysis. Different letters (a–e) indicate significant differences detected between varieties by Tukey’s honestly significant difference test, *p* < 0.05.

The expression levels of the selected candidate genes among six papaya varieties are shown in Figure 3. Significant differences in expressions were detected in all candidate genes. Among them, eight genes were related to sugar metabolism, and two were involved in VOC metabolisms. For glucose-related genes, *cpBGH3B*, *cpBGLU42,* and *cpBGLU31* were predicted to be related to the beta-glucosidase enzyme family. ‘RB1’ and ‘Holland’ had the highest expression levels of *cpBGH3B*, while there was no significant difference in the expression of *cpBGLU42* in these two papaya varieties. Instead, ‘1B’ showed the highest expression of *cpBGLU42*. ‘H13’ demonstrated the highest expression of *cpBGLU31* while ‘Skybury’ had the lowest expression. *cpRFS2* is the gene related to sucrose galactosyltransferase, and ‘Skybury’ presented a substantially higher expression of *cpRFS2*. Moreover, *cpSTP14* and *cpSTP1* are two genes related to the sugar transport protein family. ‘RB1’ and ‘Skybury’ showed higher expressions of *cpSTP14,* and ‘Holland’ had the lowest expression. While *cpSTP1* showed an opposite trend, ‘Holland’ had the highest expression of *cpSTP1*, and ‘RB1’ showed the lowest expression. For volatile metabolism-related genes, *cpGES* functioned in (E, E)-geranyl linalool synthase. ‘Skybury’ expressed significantly high *cpGES*, and ‘H13’ and ‘Holland’ had lower expressions. In addition, the differential expression levels between papaya varieties were assessed and are listed in Table S1 (Supplementary Materials). In which, ‘RB1’ was set as the control group, while the positive and negative log-fold change (log FC) values indicated the up- or downregulation of target gene expressions compared to ‘RB1’, respectively. Based on the results, most genes showed significantly different expression levels than ‘RB1’. For example, all five papaya varieties exhibited significant differential expressions of *cpGPT2*, *cpPFP*, and *cpGES*.

## 3. Discussion

Flavour is a major factor influencing consumer purchasing decision, with a drop-off in flavour quality leading to consumer dissatisfaction [16,22]. Therefore, recent breeding has concentrated on improving fruit flavour quality traits to expand the market [1,4]. Meanwhile, human flavour perception includes sugars, acids, and a group of volatile compounds as well as quantitative information from diverse sensory systems [22]. To improve the flavour of Australian papaya varieties, objective standards of good taste and aroma must be set, and the key underpinning metabolism pathways are needed for in-depth investigation. In this study, by using a combination of biochemical, sensorial and consumer acceptability evaluation, we identified the key biochemical variables which significantly correlated to consumer liking and show their application in a selective breeding program.

Papaya sweetness is mainly contributed by glucose, fructose and sucrose [4,15]. During fruit development, glucose is the predominant sugar that starts accumulating since seed maturation. When fruit is harvested and starts ripening, sucrose becomes the major sugar, which results in the sweet taste in the fruit flesh [14]. Fruit sweetness could be evaluated as the unit Degrees Brix (◦Brix), which indicates the total soluble solids in solution, which is a traditional method used by the fruit juice and fresh produce industry [4]. For papaya, Brix is considered as the percentage of glucose, fructose, and sucrose in the solution [23]. The minimum Brix level requirement for mature papaya fruit in Hawaii should be 11.5% to meet market grade [24]. Red papayas have overall higher Brix levels than yellow papayas, which may indicate that red-flesh varieties contain higher sugar contents than yellow varieties. Based on the correlation analysis between sugar determination and sensory panel outputs, the sweetness level evaluated in Brix correlated positively with the perception of sweetness by humans (*p* < 0.05). ‘Skybury’ fruit was found to be the sweetest, due to that fact of its higher sweet caramelised flavour score (74.8 ± 9.6) and sweet aftertaste score (54.8 ± 18.2). Compared to other cultivars with 19.2–64.2 for *sweet caramelised flavour* and 9.2–45.2 for *sweet aftertaste*, the lowest scores came from ‘H13’. The concentrations of three sugar components showed no significant difference among the five papaya varieties, while the percentage of glucose in total sugar was significantly different among varieties (*p* < 0.05). Glucose was, on average, 25% of total sugars in all varieties, while sucrose was approximately 40% of total sugars from our study, which were similar to those described by Nantawan et al. [19] for Australian papaya cultivars ‘Sunrise Solo’ and ‘RB2’. A previous study illustrated that each sugar type has a different contribution to the perception of flavour, and sucrose has the most contribution to the sweetness taste, while glucose sweetness is only 55 to 60% of that of fructose or sucrose and has the presence of a slight bitterness [25,26]. The correlation between sensory panel results and sugar components indicated that the percentage of glucose in total sugars was negatively correlated to the *sweetness* perception and positively correlated to *bitterness flavour* (*p* < 0.05), while sucrose showed an opposite result. In general, yellow papaya varieties ‘1B’ and ‘H13’ had lower TSS levels and higher %Glucose values, which leads to the bitterness flavour perceived by human.

A total of 14 volatiles were precisely identified from five papaya varieties through GCMS analysis. Of them, 12 volatiles have been identified in various papaya varieties from previous studies [6,8,9,10,12,27]. Among these 12 volatiles, linalool and benzyl isothiocyanate have been reported as the key impact odorants in papaya [9]. The outcome of our study is consistent with this theory; the concentrations of linalool and benzyl isothiocyanate were abundant in all varieties without statistical differences (*p* < 0.05). The remaining two compounds were citronellol and eucalyptol, which have been reported as fruit and floral constituents in grape, apple, peach, rose (*Rosa* spp.), and Tulip (*Tulipa* spp.) [28,29,30], but to the best of our knowledge, these two volatiles have not been assigned in papaya fruit. The results from the GCMS analysis indicated that yellow-fleshed variety ‘1B’ had significantly higher concentrations of linalool oxide and terpinolene than the red-fleshed variety ‘Skybury’. Terpinolene is known for its pleasant sweet-pine, citrus aroma, which is considered the major VOC responsible for the characteristic flavour of mango cultivars [31,32]. Linalool oxide has a fresh floral citrus scent and is identified in most tropical fruits [28,32,33]. Due to the low odour threshold of these two VOCs, they play key roles in ‘citrus’, ‘sweet’, and ‘floral’ aromas characteristic of papaya [33]. This also corresponded to the correlation analysis between the sensory panel results and VOCs in our study, which showed that the concentrations of linalool oxide and terpinolene were significantly positively correlated to the *citrus aroma* perceived by the trained panel (*p* < 0.05). It is also interesting to find that these two VOCs were also positively correlated to the *musty off-note aroma* but negatively correlated to the *fishy aroma*. Further investigation is needed to determine the key VOCs that lead to the production of musty off-notes and fishy aroma. The multiple linear regression model was generated using three major biochemical variables: percentage of glucose in total sugar and concentrations of linalool oxide and terpinolene, which could explain over 99% of the variability in consumer liking. However, the consumer-liking score from our study has limitations. Most of the people involved in the consumer acceptability study were from the elder age group (50–80 years old), and over 50% of them had a papaya consumption habit in which they consumed at least one papaya per month; this could lead to bias in the means of the overall liking score. Moreover, only 40 panellists were included in this study, and broader consumer groups are required to generate a more reliable linear regression model.

Moreover, the genomic-based study to identify and validate genes related to sugars and VOCs synthesis metabolism pathways is required to assist in future DNA marker development to select premium papaya cultivars that align with consumer acceptance and demand. Under such conditions, a time efficiency with a high accuracy method to evaluate the candidate genes in a large papaya population is required. NanoString nCounter technology is a simple, robust, and highly reproducible method for the quantifying expression levels of multiple genes in a single reaction [34,35]. In our study, the nCounts from nanostring were firstly normalised using reference genes, then a *t*-test was applied to determine the significant differences between samples. This comparison was allowed due to the lack of amplification steps involved during Nanostring analysis. Two new papaya varieties were included in this experiment: ‘Sunshine’ and ‘Holland’. ‘Sunshine’ is a new red-flesh papaya variety from the Australian breeding line. ‘Holland’ is also a red-flesh variety from Thailand, and this variety is also called ‘Plak Mai Lai’ in Thailand or ‘Sekaki’ elsewhere. In addition, ‘RB4’ was excluded from the gene expression analysis due to the shortage of fruit supplements.

Among the 10 candidate genes assessed, four of them were related to glucose production. *cpGPT2* is predicted to be a glucose 6-phosphate (Glc6P) transporter (GPT2), which functions in transporting Glc6p into plastids of heterotrophic tissues to start starch biosynthesis [36]. The accumulation of GPT2 is positively correlated with the amount of total soluble sugars, especially glucose [36,37]. The expression level of *cpGPT2* was significantly higher in ‘1B’ than in ‘RB1’, ‘Skybury’, and ‘H13’, which corresponded to the sugar concentration results from our biochemical evaluation, in which ‘1B’ had the highest %Glucose level. Furthermore, *cpBGH3B*, *cpBGLU42,* and *cpBGLU31* are three predicted beta-glucosidase-related genes, which have been identified to release glucose from polysaccharides [38,39]. Among them, *cpBGLU42* and *cpBGLU31* shared similar expression patterns, while the yellow-flesh varieties ‘1B’ and ‘H13’ had higher expression levels than red-flesh varieties ‘RB1’ and ‘Skybury’. *cpBGH3B* is predicted to be a beta-glucosidase BoGH3B-like gene. ‘RB1’ and ‘Holland’ had the highest expression levels of *cpBGH3B*, while the lowest expression came from ‘Sunshine’. The DE analysis also demonstrated the significant difference between ‘Sunshine’ and ‘RB1’. Beta-glucosidase BoGH3B has been identified as an enzyme involved in the xyloglucan pathway, which functions in the degradation of cellulose to glucose in peach [39,40]. However, a BoGH3B-like gene has not been enzymatically characterised to date. 

Except for glucose-related genes, *cpRFS2* is predicted to be a transglycosidase, which functions in a ping-pong reaction mechanism to catalyse the transfer of alpha-D-galactosyl-(1->3)-1D-myoinositol and sucrose to myo-inositol and raffinose [41]. Raffinose is a trisaccharide composed of galactose, glucose, and fructose. ‘Skybury’ had significantly higher expression of *cpRFS2* than other varieties. However, the sugar determination outputs from the previous experiment showed ‘Skybury’ had the lowest concentrations of glucose and fructose but the highest sucrose. This could be the result of the involvement of other enzymes which function in catalysing glucose and fructose to sucrose more actively than *cpRFS2*. In addition, *cpPFP* is predicted to be involved in the subpathway of glycolysis that synthesizes D-glyceraldehyde 3-phosphate and glycerone phosphate from D-glucose [42]. The amount of this enzyme activity was proved to be negatively correlated to the sucrose content in sugarcane [43]. In our research, ‘H13’ had the lowest expression level in *cpPFP*, while ‘Skybury’ and ‘Sunshine’ had higher expression levels. However, the sucrose content in ‘H13’ was lower than ‘Skybury’, which is opposite to the conclusion from sugarcane. Two sugar transport-related genes were also involved in this study, both of them functioned in mediating the uptake of hexoses by sugar/hydrogen symport [44]. Previous research illustrated that *cpSTP14* specifically transports glucose and galactose, and *cpSTP1* shows a significant impact on sugar uptake during plant seedling development in *Arabidopsis thaliana* [44,45,46]. The hexoses included in papaya fruit are glucose and fructose [47]. ‘RB1’ expressed significantly highest in *cpSTP14* but lowest in *cpSTP1*, while ‘Holland’ expressed lowest in *cpSTP14* but highest in *cpSTP1*. Although sugar transporter proteins are essential to plants’ sugar transport, growth, and development, the functional differentiation of these proteins in papaya is still unclear.

The remaining two genes are related to VOCs synthesis. *cpGES* is predicted to be an (E, E)-geranyl linalool synthase involved in the terpenoid biosynthesis [48]. The cooperation of (E, E)-geranyl linalool synthase initiates the catalysis of 4,8,12-trimethyltrideca-1,3,7,11-tetraene (TMTT), which is a floral odour compound identified from flowering plants such as Magnoliaceae family [48,49]. *cpBEBT* is predicted to be a benzyl alcohol O-benzoyl transferase, which is potentially involved in the production of volatile ester benzyl benzoate [50]. Benzyl benzoate is an aroma compound with a fresh floral scent identified from flowers and fruit including *Clarkia breweri,* cranberries, and papaya [50,51,52]. Both genes are related to the synthesis of floral scents, while ‘Skybury’ was significantly highly expressed in *cpGES*, and ‘H13’ was significantly highly expressed in *cpBEBT*. ‘Holland’ expressed the lowest in two genes. From sensory panel results, ‘Skybury’ had a dominating floral flavour, and ‘H13’ had high citrus and sweet fruit aromas. It is worth adding that floral aroma is a new descriptor in the sensory panel testing attributes list, and the sensory profiles of ‘Holland’ and ‘Sunshine’ are also required in the next step of research. Furthermore, the aroma compounds TMTT and benzyl benzoate have not been validated from our GCMS study and are worth adding to the future volatile compound analysis.

## 4. Materials and Methods

### 4.1. Plant Material

The fruit of five Australian commercial papaya varieties harvested from North Queensland, Australia (17.0° S, 145.4° E), were used in this study. These were the red-fleshed ‘RB1’, ‘RB4’, and ‘Skybury’, and the yellow-fleshed ‘1B’ and ‘H13’ varieties. Three biological replicates of each variety were used where a replicate was a single fruit collected from each variety at the same time and from the same field site. ‘RB1’, ‘RB4’, and ‘1B’ were collected from Lecker Farming (latitude −16.979592, longitude 145.329436); ‘Skybury’ was collected from Skybury Farmgate (latitude −17.010049, longitude 145.337257); ‘H13’ was collected from Rocky Top Farm (latitude −17.167580, longitude 145.110660). Fruit were picked from randomly selected trees at the 50–75% maturity stage [1] and stored in a temperature-controlled room at 27 °C for 24 h before being transported in a refrigerated truck to the laboratory at Griffith University Nathan Campus, Brisbane, Queensland. Fruits were subsequently stored at 23 +/− 2 °C for approximately one week until further processing.

### 4.2. Sensory Descriptive Profiling

Two sensory trials were conducted at the purpose-built sensory laboratory of the Queensland Alliance for Agriculture and Food Innovation (QAAFI) at the University of Queensland, Brisbane. Papaya fruit were taken from the Griffith University laboratory and kept at 12 °C (if already ripe), 15 °C (if nearly ripe), or at 22 °C (if largely unripe) to uniformly ripen, which was determined using the industry evaluation index [1]. On the morning of each panel trial, samples were prepared by washing and cutting each fruit in half, removing the seed and skin, and cutting one half of the flesh into cubes (~2.0 cm^3^). Cubes of fruit (~15–20 g) were dispensed into plastic cups (45 mL size), labelled with a 3-digit code, covered with clear plastic lids, and stored at room temperature (22 °C) until use. Individual fruits were used for each replicate in evaluations. The other half of each fruit was deseeded and deskinned, then blended into a puree, stored on ice, and transported to the laboratory for immediate biochemical analyses.

Conventional sensory descriptive profiling was used to evaluate the samples including a training phase with a panel of experienced tasters and the development of a lexicon and method of assessment and formal evaluation under controlled conditions. Formal evaluation sessions were conducted over 3 consecutive days, and a 10-member panel of experienced trained assessors were involved. The sensory attributes, together with their definitions and composition of the sensory reference standards, are detailed in Table 5. In each session, the panellists were asked to go through the definitions of the attributes and reassess the sensory reference standards before assessing samples. The method developed for assessment is detailed as follows: lift lid and assess aroma, take a cube of fruit to assess texture, take another cube of fruit to assess flavour and aftertaste, repeat as necessary, and cleanse between samples with water. All attributes were rated on unstructured line scales (0–100), anchored from ‘none’ to ‘high’. Data were collected electronically using the software Redjade (Redjade Software Solutions, LLC, Tragon Corporation, Redwood City, CA, USA, 2019).

### 4.3. Sugar Content Determination

Total soluble solids (TSS) were measured using a digital refractometer and expressed as ◦Brix [4]. To determine the concentrations of the three main soluble sugars, samples (from Section 4.1) were removed from the seed and skin, using the flesh only. Flesh was then flash-frozen in liquid N_2_ and finely ground by mortar and pestle. Subsequently, 100 mg of ground sample was weighed and transferred to a sterile 2 mL microcentrifuge tube with 500 μL of 80% ethanol. The mixture was then incubated at 78 °C for 10 min and vortexed for 30 s. After, samples were centrifuged at 13,000 rpm (17,000 rcf) for 5 min at 4 °C. The supernatant was transferred to a new 1.5 mL microcentrifuge tube and the ethanol was evaporated overnight at room temperature (23 ± 2 °C) to obtain the sugar extracts. Glucose, fructose, and sucrose concentrations per 100 mg of sample were then determined using a microplate enzymatic assay described by Velterop and Vos [53].

### 4.4. Volatile Compound Analysis

A deseeded and deskinned flesh sample from each fruit, as listed Section 4.1, was homogenised into a puree using a blender and transferred into a sealed 15 mL sterile falcon tube; the tubes were flash-frozen in liquid N_2_ and then sent to ACS laboratories (Kensington, Victoria, Australia; www.acslab.com.au, accessed on 18 August 2020) on dry-ice for volatile compound analysis. The method was developed by ACS lab and is as follow: Samples were accurately weighed into 15 mL polypropylene tubes using analytical balance, and sodium sulphate was then added for extraction purposes. The organic layer obtained after the extraction was then spiked with an internal standard (nonane: 0.858 mg/L) and transferred to the 20 mL headspace vial. Reference standards were added and included α-pinene, β-pinene, β-myrcene, 3-carene, p-cymene, D-limonene, eucalyptol, linalool oxide, linalool, terpinolene, citronellol, naphthalene, citral, and benzyl isothiocyanate, which were provided by ACS lab.

Gas chromatography mass spectrometry (GCMS) analysis was performed using a 7890A gas chromatography coupled with a 5975C mass selective detector (MSD) (Agilent Technologies, Santa Clara, CA, USA). A DB-5MS 30 m × 0.25 mm × 0.25 μm column (Agilent J&W Columns) was used for chromatographic separation, and MSD was run in the SIM/Scan Mode. All the GCMS data, including concentrations of each reference standard and VOC from each papaya sample, were collected by ACS lab.

### 4.5. Consumer Acceptability Study

Individuals from the local community in Mareeba, Australia, were involved in the consumer acceptability survey to score the overall acceptance of the five papaya varieties (‘RB1’, ‘RB4’, ‘Skybury’, ‘1B’, and ‘H13’). The panellists’ (*n* = 40; 30 female, 10 male) ages were 10% 20–40 years, 25% 40–50 years, and 65% 50–80 years with differing papaya consumption habits (12.5% never, 40% monthly, 37.5% weekly, and 10% daily). A questionnaire was designed with a score of overall liking rating from “extremely disliked” (1) to “extremely liked” (9). Papaya fruits were washed, cut into cubes (~2.0 cm^3^), dispensed into plastic cups with lids, labelled with a random number sequence, stored at 10 °C overnight and offered for tasting at RT the following day.

### 4.6. Data Analysis

The data obtained from the sensory panel analysis, sugar and volatile evaluation, and consumer acceptability test were analysed by one-way analysis of variance (ANOVA) followed by Tukey’s honestly significant difference test with a 5% significance level using XLSTAT (version 2021.5). Subsequently, unsupervised principal component analysis (PCA) was performed on the sample mean scores to visualise clustering of papaya based on the sensory attributes. Correlation analysis between means of consumer-liking scores and sensory descriptors as well as between the most significant sensory attributes and biochemical variables, was determined by Pearson correlation with a 5% significance level. Multiple linear regression models were developed using XLSTAT (version 2021.5) to directly compare and find associations between the related biochemical variables and consumer liking, and an equation was generated to predict the consumer-liking score using the most influential biochemical variables.

### 4.7. Differential Gene Expression Analysis

RNA was extracted from 50 mg samples from each of six papaya varieties (i.e., ‘RB1’, ‘1B’, ‘H13’, ‘Skybury’, ‘Sunshine’, and ‘Holland’) using the NucleoZol kit protocol (Macherey-Nagel Inc., Allentown, PA, USA). ‘RB1’, ‘1B’, and ‘Holland’ were collected from Lecker Farming (latitude -16.979592, longitude 145.329436); ‘Skybury’ was collected from Skybury Farmgate (latitude −17.010049, longitude 145.337257); ‘H13’ and ‘Sunshine’ were collected from Rocky Top Farm (latitude −17.167580, longitude 145.110660). Three biological replicates of each variety were applied. NanoString Elements Tagset (NanoString Technologies, Seattle, WA, USA) was custom designed to screen gene sequence targets for differential expression among papaya varieties through high throughput plate assays in this research. This panel contained a total of 13 papaya genes of which three were reference genes and 10 were papaya flavour-related genes mined from the NCBI databases (https://www.ncbi.nlm.nih.gov, accessed on 16 April 2021) using the following keywords: glucose, fructose, sucrose, sugar transport, linalool, and benzyl (Table 6). RNA samples were prepared by adding 7 μL of sample to a probe pool and hybridising at 65 °C for 20 h. Samples were processed on the NanoString Prep Station and cartridges were scanned by the nCounter Digital Analyser for digital counting of molecular barcodes corresponding to each target at 555 fields of view. Data quality control (QC) and normalisation were undertaken with nSolver 4.0 (NanoString Technologies, WA, USA). Raw gene expression data were normalised against a set of six positive and six negative controls to account for background noise and platform-associated variation. Data were filtered to exclude genes expressed below a minimum detection threshold of 20. Reference gene normalisation was performed using the geNorm algorithm based on a pairwise comparison of the reference genes [54]. ANOVA followed by Tukey’s honestly significant difference test with a 5% significance level was then performed on the normalised nCounts data using XLSTAT (version 2021.5). Differential expression analysis was performed among the varieties using the “NanoStringDiff” package (version 1.24.0) using the R language for statistical analysis (version 4.0.3) [55].

## 5. Conclusions

In this study, we investigated the flavour profiles of major Australian papaya varieties. In general, red-flesh varieties ‘RB1’, ‘RB4’, and ‘Skybury’ had higher sugar content levels than yellow-flesh varieties ‘1B’ and ‘H13’. On the contrary, yellow papaya had higher concentrations of volatile compounds linalool oxide and terpinolene than red papaya. These two volatiles demonstrated high correlation coefficient values (0.916 and 0.946, respectively) to the citrus aroma from sensory descriptive profiling. In addition, the percentage of glucose in total sugar, and the concentrations of linalool oxide and terpinolene were identified as the key drivers of consumer liking. The analysis of gene expression revealed that 10 differentially expressed genes functioned in sugar and volatile metabolisms. Nanostring experiments verified that those genes were differentially expressed among the six papaya varieties. Key genes, such as *cpGPT2*, *cpBGLU42*, and *cpBGLU31*, showed higher expression levels in ‘1B’ than red papaya varieties, resulting in the accumulation of glucose and were possibly responsible for the higher glucose level in ‘1B’.

To sum up, this study involves a basic understanding of what constitutes flavour and aroma preference in Australian papaya varieties and also provides approaches to discovering the genes that are significantly associated with key aromas and flavours. The future directions of this study should be focused on discovering more biosynthesis metabolism pathways underpinning preferable papaya flavour, thus contributing to the future breeding, branding, and marketing of premium papaya cultivars.

## Figures and Tables

**Table 1:** Sweetness characteristics from five papaya varieties.

Variety	Glucose (mg/g FW)	Fructose (mg/g FW)	Sucrose (mg/g FW)	Glucose (%, *w*/*w*) *	Fructose (%, *w*/*w*)	Sucrose (%, *w*/*w*)	TSS (◦Brix) *
RB1	18.1 ± 6.8	32.4 ± 3.4	37.1 ± 2.5	20.6 ± 0.02 ab	36.8 ± 0.02	42.5 ± 0.02	11.1 ± 0.7 b
RB4	17.8 ± 6.8	31.5 ± 3.4	36.6 ± 2.4	20.7 ± 0.01 ab	36.6 ± 0.02	42.7 ± 0.03	10.6 ± 0.7 bc
Skybury	15.0 ± 6.5	29.0 ± 3.3	37.4 ± 2.4	18.4 ± 0.02 b	35.6 ± 0.03	46.0 ± 0.02	12.8 ± 1.3 a
1B	20.7 ± 9.5	28.1 ± 7.4	33.3 ± 9.3	24.1 ± 0.05 a	34.0 ± 0.01	42.0 ± 0.05	9.5 ± 0.7 c
H13	22.6 ± 4.9	32.1 ± 3.9	36.5 ± 2.7	24.4 ± 0.03 a	35.2 ± 0.01	40.4 ± 0.03	9.2 ± 0.9 c
*p*-Value	0.148	0.519	0.245	0.008	0.116	0.144	<0.0001

Different letters (a–c) indicate significant differences detected between varieties by Tukey’s honestly significant difference test, *p* < 0.05. * Significance was detected; FW = fruit weight; %, *w*/*w* = percentage of target sugar level in the total sugar amount, weight/weight).

**Table 2:** Concentrations of identified volatiles in five papaya varieties after GCMS analysis.

Volatile Identity	Classification	Concentration (mg/kg) *					
		RB1	RB4	Skybury	1B	H13	*p*-Value
Linalool oxide	Monoterpene	0.087 b	0.108 ab	0.031 b	0.289 a	0.193 ab	0.013
Linalool	Monoterpene	0.016	0.029	0.004	0.098	0.024	0.078
Terpinolene	Monoterpene	0.017 bc	0.016 abc	0.002 c	0.071 a	0.051 ab	0.006
α-Pinene	Monoterpene	0.003	-	0.040	-	0.008	0.441
β-Pinene	Monoterpene	0.004	0.001	0.014	0.002	0.003	0.397
β-Myrcene	Monoterpene	0.009	0.002	0.004	-	0.002	0.719
3-Carene	Monoterpene	<0.001	-	0.016	-	0.002	0.413
p-Cymene	Monoterpene	<0.001	<0.001	0.001	<0.001	<0.001	0.312
D-Limonene	Monoterpene	0.002	0.003	0.017	0.003	0.003	0.297
Eucalyptol	Monoterpene	0.063	0.030	0.015	0.052	0.014	0.065
Citronellol	Monoterpene	0.025	0.019	0.038	0.026	0.027	0.137
Citral	Monoterpene	0.079 ab	0.038 ab	0.059 ab	0.116 a	0.046 b	0.035
Benzyl isothiocyanate	Benzene	0.201	0.269	0.046	0.198	0.121	0.267
Naphthalene	Benzene	0.343	0.329	0.354	0.382	0.335	0.447

* Mean concentration was expressed as mg nonane equivalents per kg papaya sample. Different letters (a–c) indicate significant differences detected between varieties by Tukey’s honestly significant difference test, *p* < 0.05.

**Table 3:** Correlation between consumer-liking score and sensory descriptors.

Sensory Descriptor	Consumer Liking
*sweet caramelised flavour*	**0.593**
*sweet aftertaste*	**0.542**
*velvety*	**0.302**
*fishy aroma*	**0.286**
*floral flavour*	**0.223**
*flavour intensity*	0.119
*juiciness*	0.083
*resistance*	0.044
*dissolving*	−0.060
*metallic aftertaste*	−0.121
*sweet fruit aroma*	**−0.166**
*musty off-note aroma*	**−0.205**
*fibrous*	**−0.261**
*bitter aftertaste*	**−0.298**
*bitterness flavour*	**−0.321**
*musty flavour*	**−0.325**
*aroma intensity*	**−0.360**
*citrus aroma*	**−0.441**

Values in bold are significantly different from 0 (*p*-value < 0.05).

**Table 4:** Correlation between target sensory descriptors and biochemical compounds.

(a) Correlation between target sensory descriptors and sweetness-related variables						
Variables	*Sweet Aftertaste*	*Sweet Caramelised Flavour*	*Floral Flavour*	*Bitter Aftertaste*	*Bitterness Flavour*	*Musty Flavour*
TSS	**0.797**	**0.782**	0.325	**−0.474**	**−0.436**	**−0.565**
Glucose	−0.298	−0.323	−0.156	**0.372**	**0.431**	**0.452**
Fructose	0.087	0.060	0.071	0.075	0.143	0.179
Sucrose	0.263	0.222	0.178	−0.107	−0.160	−0.225
%Sucrose	0.258	0.265	0.118	−0.319	**−0.417**	**−0.504**
%Glucose	**−0.453**	**−0.463**	−0.226	**0.444**	**0.504**	**0.540**
(b) Correlation between target sensory descriptors and volatile-related variables						
Variables	*citrus aroma*	*fishy aroma*	*aroma intensity*	*musty off-note aroma*	*sweet fruit aroma*	
Naphthalene	0.083	0.217	0.025	−0.058	−0.275	
Citral	0.209	0.151	0.215	0.186	−0.016	
Eucalyptol	−0.023	0.167	0.014	−0.121	0.198	
p-Cymene	−0.475	0.432	−0.594	−0.462	−0.185	
D-Limonene	−0.375	0.297	−0.395	−0.286	−0.164	
α-Pinene	−0.313	0.181	−0.369	−0.274	−0.102	
3-Carene	−0.343	0.245	−0.395	−0.286	−0.125	
β-Pinene	−0.346	0.209	−0.377	−0.279	−0.113	
Citronellol	−0.468	0.365	−0.278	−0.329	0.017	
Benzyl isothiocyanate	0.285	−0.177	0.052	0.074	0.038	
Linalool	**0.722**	**−0.614**	**0.649**	0.400	0.507	
Terpinolene	**0.946**	**−0.828**	**0.850**	**0.662**	0.275	
Linalool oxide	**0.916**	**−0.768**	**0.799**	**0.631**	0.265	
β-Myrcene	−0.439	0.252	−0.348	−0.411	0.127	

Values in bold are significantly different from 0 (*p*-value < 0.05).

**Table 5:** Sensory panel testing attributes.

Attribute	Definition
**Aroma**	
*overall aroma intensity*	The overall intensity of the sample aroma
*sweet fruit*	An aroma of fresh sweet fruit such as honeydew melon or mango
*musty off-note*	An aroma of ripe rock melon, over-ripe fruit, eggy, sulphurous
*fishy*	An aroma of tuna, fishy, or seaweed
*citrus*	An aroma of citrus peel or juice
**Texture**	
*resistance*	The degree to which the sample resists initial bite, firmness
*juiciness*	The degree to which liquid is released upon mastication
*dissolving*	The degree to which the sample dissolves/disintegrates in the mouth
*velvety*	The smoothness of the sample (lack of particles/grit)
*fibrous*	The presence of fibrous pieces, debris in the sample
**Flavour**	
*flavour intensity*	The overall flavour intensity of the sample
*sweet caramelised*	A flavour associated with cooked sweet potato/carrot, sweet melon with caramel notes
*bitterness*	A bitter flavour
*musty*	A flavour of over-ripe rockmelon with skin, stale
*floral*	A flavour of floral notes (jasmine flower)
**Aftertaste**	
*bitter*	A bitter aftertaste
*sweet*	A sweet aftertaste
*metallic*	A metallic aftertaste

**Table 6:** Candidate gene list for Nanostring analysis.

Gene ID	Accession ID	Description
*SAND*	JQ678783	*Carica papaya* SAND family protein-like (SAND) mRNA
*TBP2*	JQ678779.1	TATA-binding protein 2
*CYP*	JQ678769.1	Cyclophilin
*cpGPT2*	XM_022031675.1	PREDICTED: *Carica papaya* glucose-6-phosphate/phosphate translocator 2, chloroplastic (LOC110806751), mRNA
*cpBGH3B*	XM_022036929.1	PREDICTED: *Carica papaya* beta-glucosidase BoGH3B-like (LOC110810687), mRNA
*cpBGLU42*	XM_022038006.1	PREDICTED: *Carica papaya* beta-glucosidase 42-like (LOC110811486), mRNA
*cpBGLU31*	XM_022053230.1	PREDICTED: *Carica papaya* beta-glucosidase 31-like (LOC110822982), mRNA
*cpRFS2*	XM_022037673.1	PREDICTED: *Carica papaya* probable galactinol--sucrose galactosyltransferase 2 (LOC110811248), transcript variant X3, mRNA
*cpPFP*	XM_022039277.1	PREDICTED: *Carica papaya* pyrophosphate-fructose 6-phosphate 1-phosphotransferase subunit beta (LOC110812486), mRNA
*cpSTP14*	XM_022044378.1	PREDICTED: *Carica papaya* sugar transport protein 14 (LOC110816261), mRNA
*cpSTP1*	XM_022055661.1	PREDICTED: *Carica papaya* sugar transport protein 1-like (LOC110825204), mRNA
*cpGES*	XM_022046578.1	PREDICTED: *Carica papaya* (E, E)-geranyllinalool synthase-like (LOC110817861), mRNA
*cpBEBT*	XM_022055614.1	PREDICTED: *Carica papaya* benzyl alcohol O-benzoyltransferase-like (LOC110825152), mRNA

## Data Availability

The data presented in this study were deposited to Zenodo and are available at doi:10.5281/zenodo.6583115.

## Supplementary Materials

The following supporting information can be downloaded at: https://www.mdpi.com/article/10.3390/ijms23116313/s1.
