# Supplementary material for: Biochemical, Sensory, and Molecular Evaluation of Flavour and Consumer Acceptability in Australian Papaya (Carica papaya L.) Varieties
Source: Int J Mol Sci. 2022 Jun 5;23(11):6313. doi: 10.3390/ijms23116313 (PMC9181177; doi:10.3390/ijms23116313)
Supplement: Supplementary file 1 [file ijms-23-06313-s001.zip › ijms-1748402-supplementary.pdf]

Table S1: Differential expression of flavour related genes in papaya varieties compared to RB1

ID	logFC	pvalue	qvalue	comparison
cpGPT2	1.684576	0	0	1B_vs_RB1
cpGPT2	0.846062	0.000204	0.00039	H13_vs_RB1
cpGPT2	2.780658	0	0	Holland_vs_RB1
cpGPT2	1.032886	2.37E-06	4.99E-06	Skybury_vs_RB1
cpGPT2	0.824507	0.000164	0.000345	Sunshine_vs_RB1
cpBGH3B	-1.42285	0	0	1B_vs_RB1
cpBGH3B	-0.90815	1.26E-10	3.77E-10	H13_vs_RB1
cpBGH3B	-0.12824	0.331677	0.464348	Holland_vs_RB1
cpBGH3B	-0.93366	2.17E-11	5.7E-11	Skybury_vs_RB1
cpBGH3B	-2.41352	0	0	Sunshine_vs_RB1
cpBGLU42	0.399146	0.001514	0.002649	1B_vs_RB1
cpBGLU42	0.102445	0.413459	0.542665	H13_vs_RB1
cpBGLU42	0.098317	0.436592	0.573027	Holland_vs_RB1
cpBGLU42	-0.09616	0.44855	0.554091	Skybury_vs_RB1
cpBGLU42	0.116872	0.352137	0.434993	Sunshine_vs_RB1
cpBGLU31	0.965393	3.11E-14	8.16E-14	1B_vs_RB1
cpBGLU31	1.464585	0	0	H13_vs_RB1
cpBGLU31	-1.08281	3.33E-16	9.99E-16	Holland_vs_RB1
cpBGLU31	-2.9556	0	0	Skybury_vs_RB1
cpBGLU31	-0.02487	0.844124	0.984811	Sunshine_vs_RB1
cpRFS2	-1.02905	3.33E-15	1.17E-14	1B_vs_RB1
cpRFS2	-0.54164	2.73E-05	5.73E-05	H13_vs_RB1
cpRFS2	-0.02763	0.820068	0.956746	Holland_vs_RB1
cpRFS2	1.353015	0	0	Skybury_vs_RB1
cpRFS2	0.138299	0.287098	0.376816	Sunshine_vs_RB1
cpPFP	0.291127	0.041516	0.062274	1B_vs_RB1
cpPFP	-0.74186	2.14E-07	5E-07	H13_vs_RB1
cpPFP	0.402175	0.004803	0.00917	Holland_vs_RB1
cpPFP	0.487365	0.000649	0.001239	Skybury_vs_RB1
cpPFP	0.517103	0.000287	0.000548	Sunshine_vs_RB1
cpSTP14	-2.26699	0	0	1B_vs_RB1
cpSTP14	-1.42881	0	0	H13_vs_RB1
cpSTP14	-2.7633	0	0	Holland_vs_RB1
cpSTP14	-0.64505	9.1E-12	2.73E-11	Skybury_vs_RB1
cpSTP14	-1.2842	1	1	Sunshine_vs_RB1
cpSTP1	1.145416	2.44E-09	5.12E-09	1B_vs_RB1
cpSTP1	0.188283	0.355882	0.498235	H13_vs_RB1
cpSTP1	2.281872	0	0	Holland_vs_RB1
cpSTP1	1.684405	0	0	Skybury_vs_RB1
cpSTP1	0.913392	2.26E-06	5.94E-06	Sunshine_vs_RB1
cpGES	-0.31184	0.032564	0.052604	1B_vs_RB1
cpGES	-2.90211	0	0	H13_vs_RB1
cpGES	-3.54617	0	0	Holland_vs_RB1

cpGES	2.124663	0	0	Skybury_vs_RB1
cpGES	1.471937	0	0	Sunshine_vs_RB1
cpPBEBT	-0.01126	1	1	1B_vs_RB1
cpBEBT	0.501456	0.000804	0.001408	H13_vs_RB1
cpBEBT	-1.31332	0	0	Holland_vs_RB1
cpBEBT	-0.37873	0.010915	0.019101	Skybury_vs_RB1
cpBEBT	-1.18032	4.33E-15	1.82E-14	Sunshine_vs_RB1
